# Epidemiological and Inducible Resistance in Coagulase Negative Staphylococci

**DOI:** 10.5539/gjhs.v8n4p109

**Published:** 2015-07-30

**Authors:** Shadieh Abdollahi, Rashid Ramazanzadeh, Zahra Delami Khiabani, Enayat Kalantar

**Affiliations:** 1 Department of Microbiology, Islamic Azad University, Zanjan Branch, Zanjan, Iran; 2 Cellular & Molecular Research Center, Kurdistan University of Medical Sciences, Sanandaj, Iran; 3 Department of Ginetic, Islamic Azad University, Zanjan Branch, Zanjan, Iran; 4 Department of Microbiology and Immunology, School of medicine, Alborz, University of Medical Sciences, Karaj, Iran

**Keywords:** coagulase negative staphylococci, Multiplex PCR, ERIC–PCR, resistance

## Abstract

**Background and Objectives:**

Coagulase negative staphylococci (CNS) are potential pathogens with the increased use of implants in hospitals. Macrolide, lincosamide and streptogramin B (MLSB) are used in the treatment of staphylococcal infections. The aim of this study was to molecular detection of inducible clindamycin resistance and genetic pattern in CNS isolates and their transmission between hospitals.

**Materials and Methods:**

110 CNS strains, isolated from hospitalized patients in the intensive care unit and infectious wards of Besat and Toohid hospitals, Sanandaj. Methicillin resistance was done by agar screen test and the resistance inducible Clindamycin by the D-Test. Multiplex PCR was performed, using primers specific for *erm* (A, B, C, *and TR*) genes. Diversity of strains was determined by ERIC–PCR technique based on the similarities between DNA fingerprints by using Jaccards coefficient in the SAHN program of the NTSYS-pc software.

**Results:**

Of the 110 isolates, 64(58.2%) were methicillin -resistant CNS (MRCNS), 48(43.6%) were resistant to erythromycin (ERCNS). Out of 48 Erythromycin-resistant strains 5 (10.4%) were iMLS_B_ phenotypes that 4 isolates showed genes *erm* by Multiplex PCR. The ERIC–PCR profiles allowed typing of the 110 isolates into 90 ERIC-types which were grouped into fourteen main clusters (C1–C14).

**Conclusion:**

The results of this study also showed that most of CNS isolated produced different genomic fingerprint patterns, therefore, source of infection is differen t.

## 1. Introduction

Coagulase negative staphylococci (CNS), now considered to be as significant potential pathogens with the increased use of implants in hospitals (Azuka, & Idahosa, 2013). However, with upraise use modern medicine that show dependence to medical device materials to production biofilm also CNS identified mainly to cause nosocomial infections (Azuka, & Idahosa, 2013; Dryden, 2010; Caseya, Lambertb, & Elliotta; 2007), Most of diseases or infections assumed by CNS are significant as a result of hospitalization (Dryden, 2010). CNS have historically been more resistant to antimicrobials including the -lactam antibiotics, than *S. aureus* (Longauerova, 2006).

Antibiotics MLS_B_ group (Macrolides, Lincosamides and StreptograminB) are inhibit bacterial protein synthesis by binding to the 23srRNA. Among this group of drugs, Clindamycin is a good substitute to treat soft tissue infections by both methicillin-resistant Staphylococcus and methicillin- sensitive Staphylococcus infections. Frequent use of these drugs increases the resistance, especially induction resistance, and consequently failure of treatment with clindamycin (El-Mahdy, Abdalla, El-Domany, & Snelling, 2010; Fasih, Irfan, Zafar, Khan, & Hasan, 2010; Mohanasoundaram, 2011). Four major classes of the *erm* genes including [*erm*(A), *erm*(B), *erm*(C) and *erm*(TR)], are known to be responsible for the MLS_B_ resistance in staphylococci, and can provide either constitutive resistance (cMLS_B_) or inducible resistance (iMLS_B_) (Fasih, Irfan, Zafar, Khan, & Hasan, 2010; Mohanasoundaram, 2011; Shantala, Shetty, Rao, & Nagarathnamma, 2011).

Epidemiological studies survey the time and distribution of infectious diseases and effort to determine the factors influencing prevalence (Fiebelkorn, Crawford, McElmeel, & Jorgensen, 2003). Repetitive extragenic palindromic elements-polymerase chain reaction (REP-PCR) is method that produces the DNA fingerprint which discriminates between bacterial strain, and have sporadically been used to characterize (Generscha, & Ottenb, 2003). There are three main sets of repetitive DNA elements used for typing purposes. The repetitive extragenic palindromic (REP), enterobacterial repetitive intergenic consensus (ERIC) and BOX elements including of differentially subunits, boxA, boxB, and boxC (Bou, Deminguez, Quereda, & Martinez-Beltran, 2000). Rep-PCR has been identified as a simple PCR-based technique with the following characteristics: (i) low cost, (ii) high discriminatory power, (iii) suitable for a high-throughput of strains, and (iv) considered to be a trusty tool for classifying and typing a wide range of Gram-negative and several Gram positive bacteria (Bou, Deminguez, Quereda & Martinez-Beltran, 2000; Krizova, Spanova, & Rittich, 2008; Lima, Guidelli, Abreu, & Lemos, 2002; Lisek, Paszt, Oskiera, Trzcinski, Bogumil, Kulisiewicz, & Malusa, 2011; Lisek, Paszt, Oskiera, Trzcinski, Bogumil, A, Kulisiewicz, A, & Malusa, 2011; Soltysik, Bednarek, Loch, Galka, & Sypniewski, 2011; Ishii & Sadowsky, 2009).

The aim of this study examined PCR-amplified enterobacterial repetitive intergenic consensus (ERIC -PCR) analysis on Coagulase negative staphylococci, comparing the genetic relationship of different isolates, and also molecular detection of inducible clindamycin resistance. These studies are essential to examine the prevalence of Antibiotic-resistant strains and also source of coagulase negative staphylococci or infectious origin and their dissemination, and provide scientific basis for human disease control and warning.

## 2. Materials and Methods

**Collection and culture of isolates.** Totally 110 Coagulase negative staphylococci isolated from hospitalized patients in the intensive care unit and infectious wards of Besat and Toohid hospitals in Sanandaj. In this study 23 samples were collected in Besat hospital, and 87 samples were from Toohid hospital. Sampling was performed with sterile swab from the throat (29.1%) and nose (70.9%). The isolates were sub cultured on to blood agar and were incubated at 37°C overnight before they were used so were identified by using usual microbiological methods include colonial morphology, Gram stain, negative mannitol test and a test tube coagulase reaction, susceptibility to bacitracin and novobiocin (Pal, Sharma, Sharma, & Vyas, 2010; Moosavian, & Darban, 2010; Moosavian & Wadowsky, 2010). All the isolates were suspended in 20% glycerol stock and kept at -70°C for long-term storage (Varges, Penna, Martins, Martins, & Lilenbaum, 2009).

**Antibiotic susceptibility test.** Susceptibility to antimicrobial agents was performed according to Clinical and Laboratory Standards Institute (CLSI) guidelines. Antibiotic disc used were erythromycin (15 μg) (Franklin, Matthew, Karen, Michael, George, Dwigh, & et al, 2010).

**Methicillin resistance.** To identify strains of methicillin-resistant Coagulase negative staphylococci used specific medium and added Mueller Hinton agar medium, 4% NaCl and 6 mg/L oxacillin. Coagulase negative staphylococci strains were cultured and incubated at temperatures of 35°C for 24 hours. The growth more than one colony was showed resistance to methicillin (Franklin, Matthew, Karen, Michael, George, Dwigh, & et al, 2010).

**Figure 1:**
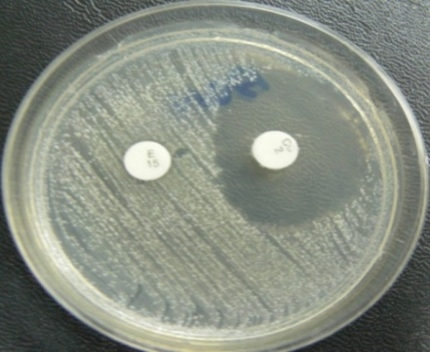
This figure shows the phenotype iMLSB observed during D-Test, E (15 μg); erythromycin, CC (2 μg); clindamycin

**Detection of inducible resistance *erm* gene expression**. Inducible Clindamycin resistance was performed according to CLSI guidelines by using D -Test method. A 0.5 McFarland equivalent suspension of organisms was inoculated onto a Mueller - Hinton agar (MHA) plate, the ER (15 μg) disk was placed 15-26 mm (edge to edge) apart from CL (2 μg) disk on MHA. Plates were surveyed after 16-18 hours of incubation at 35 °C (Figure 1) (Franklin et al, 2010; Schreckenberger, Ilendo, & Ristow, 2004).

**Figure 2:**
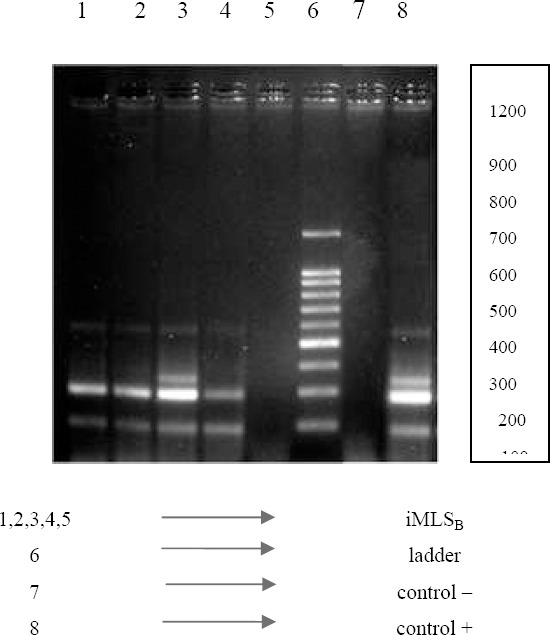
Electrophoresis Multiplex PCR product for *erm* genes

**DNA extraction.** Genomic DNA extraction of Coagulase negative staphylococci strains were performed by use of Cinna Pure kit (Cinagen, Iran).

**Multiplex PCR for gene *erm*.** The specific primers for *erm* genes were obtained are shown in Table 1 (Matthew, Sullivan, Cai, Kong, Zeng, & Gilbert, 2006).

**Table 1:** Primers used in Multiplex PCR (Matthew, Sullivan, Cai, Kong, Zeng, & Gilbert, 2006)

Gene	Primer: sequence	Size
*ermA*	ermA/TRSb: 5’ TCA GGA AAA GGA CAT TTT ACC3’	**118 bp**
	ermAAb: 3’ATA TAG TGG TGG TAC TTT TTT GAG C5’	
*ermB*	ermBAb: 5’CGA TAT TCT CGA TTG ACC C3’	**228 bp**
	ermBSb: 3’GGT AAA GGG CAT TTA ACG AC5’	
*ermC*	ermCab: 5’ TAGCAAACCCGTATTCCACG3’	**495 bp**
	ermCsb: 3’CTTGTTGATCACGATAATTTCC5’	
*ermTR*	ermTRAb: 5’AAA ATA TGC TCG TGG CAC3’	**286 bp**
	ermA/TRSb: 3’TCA GGA AAA GGA CAT TTT ACC5’	

Multiplex PCR was performed in final volume of 20 μl by DFS Master Mix Kit (Cinagen) including Taq polymerase enzyme, MgCl2, dNTP, (NH_4_)_2_SO_4_, TrisHCl, Tween – 20. Reaction mixtures consisted of 12.5 μl Master Mix, 1 μl MgCl_2_, 0.25 μl of each primer, 2 μl Distilled water and 1μl DNA template. Cycling conditions were Primary denaturation 94°C for 4 min, Denaturation 94°C for 30 s, Annealing 52 ºC for 30 s’ Extension 72 ºC for 1 min, then 30 cycle, followed by a Final Extension 72 ºC for 5 min (Matthew, Sullivan, Cai, Kong, Zeng, & Gilbert, 2006).

**Molecular typing by REP-PCR.** The Rep primers were ERIC lR: 5’-ATG TAA GCT CCT GGG GAT TCA C-3’and ERIC2: 5’-AAG TAA GTG ACT GGG GTG AGC G -3’.

Rep-PCR was performed in final volume of 25 μl by DFS Master Mix Kit PCR (BIORON) including Taq polymerase enzyme, MgCl_2_, dNTP, (NH_4_)_2_SO_4_, TrisHCl, Tween – 20. Reaction mixtures consisted of 12.5 μl Master Mix, 1 μl MgCl_2_, 1 μl of primer F, 1 μl of primer R, 7.5 μl Distilled water and 2μl DNA template. Cycling conditions were Primary denaturation 95 ºC for 2 min, Denaturation 92 ºC for 30 s, Annealing 50 ºC for 1 min, Extension 65 ºC for 8 min, then 35 cycle, followed by a Final Extension 65 ºC for 8 min.

**Electrophoresis.** Agarose gel electrophoresis was done by 1.5% (0.6 g of agarose (Neda Fan Rah, Iran) in 40 cc of 0.5X TAE buffer). After solidification and removing of the comb, 4µl PCR products with 2µl buffer loading loaded onto the gel and electrophoresis were performed for 1 h at 50 voltages. After staining with ethidium bromide (BIORON 10^mg^/_ml_), gels were scanned using device UV translluminator and Marker of Standard Molecular Marker 100 bp DNA Ladder (BIORON) was used to determine the size (Ramazanzadeh, Zamani, & Zamani, Sa, 2013).

**Computer-Assisted ERIC-PCR DNA Fingerprint Analysis.** Data matrix was formed based on presence or absence of bands and analyzed using the NTSYS-pc software (version 2.02 K, Applied Biostatistics, Inc, NY, USA). Dendrograms of dissimilarity were produced for every case. The similarity between the strains was determined on the basis of the Jaccard similarity. The dendrogram was produced on the basis of the averaged similarity of the matrix with the use of the algorithm of the Unweighted Pair-Group Method (UPGMA) in the SAHN program of the NTSYS-pc software. This method has been used to show relations between similar groups (Gadepalli, Dhawan, Mohanty, Kapil, Das, & Chaudhry, 2006).

## 3. Results

The total 110 Coagulase negative staphylococci isolates was collected 23(20.9%) specimens from Beast hospital and 87(79.1%) specimens from Toohid hospital which are shown in Table 2. The antimicrobial susceptibility of 110 Coagulase negative staphylococci isolates was determined in vitro (Table 3). Of the 110 isolates, 64(58.2%) methicillin -resistant CNS (MRCNS) and 46(41.8%) methicillin-sensitive CNS (MSCNS), a total of 110 CNS isolates were found 48(43.6%) to be resistant to erythromycin (ERCNS) and were tested for inducible resistance by double disk approximation test (D-Test). Out of 48 Erythromycin-resistant strains 5 (10.4%) were iMLS_B_ phenotypes 4 (80%) iMLS B phenotypes were observed to be methicillin-resistant.

**Table 2:** Dispersal 110 Coagulase negative staphylococci isolated from hospitalized patients

	Beast hospital		Toohid hospital	
	
	care unit ward	infectious ward	care unit ward	infectious ward
**Nose**	13(11.8%)	2(1.8%)	30(27.3%)	33(30%)
**throat**	7 (6.4%)	1 (0.9)	15(13.6%)	9 (8.2%)
**Total**	20(18.2%)	3(2.7%)	45(40.9%)	42(38.2%)

**Table 3:** Antimicrobial susceptibility of CNS isolates

	Beast hospital		Toohid hospital	
	
	Resistant	Susceptible	Resistant	Susceptible
**methicillin**	13(11.8 %)	10(9.1%)	51(46.4%)	36(32.7%)
**Erythromycin**	7(6.4%)	16(14.5%)	41(37.3%)	46(41.8%)
**cMLS_B_**	6 (12.5%)	-	29(60.4%)	-

cMLSB: constitutive resistance (Clindamycin resistance, erythromycin resistance).

Genetic studies conducted on the erythromycin-resistant isolates, searching for genes *erm*, Among the iMLS_B_ phenotypes 5 isolates, The *erm(A)* gene was detected in 80% (4/5) of the these isolates, *erm(B)* in 80% (4/5), *erm(C)* in 80% (4/5), and *erm(TR)* in 20% (1/5) isolates.

**Figure 3:**
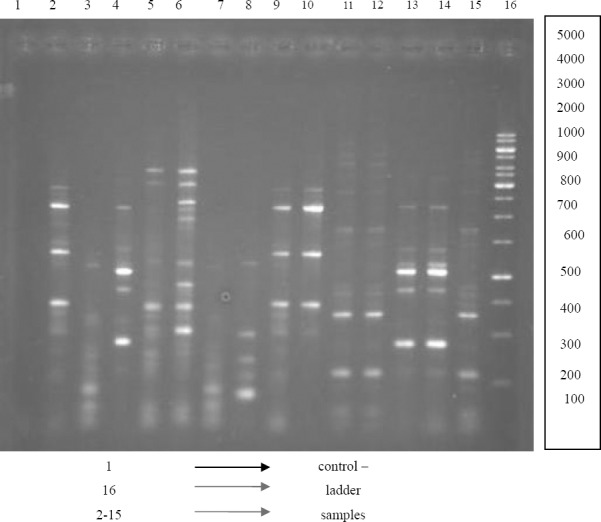
Genomic DNA fingerprinting patterns from strains CNS generated by ERIC-PCR

**Figure 4:**
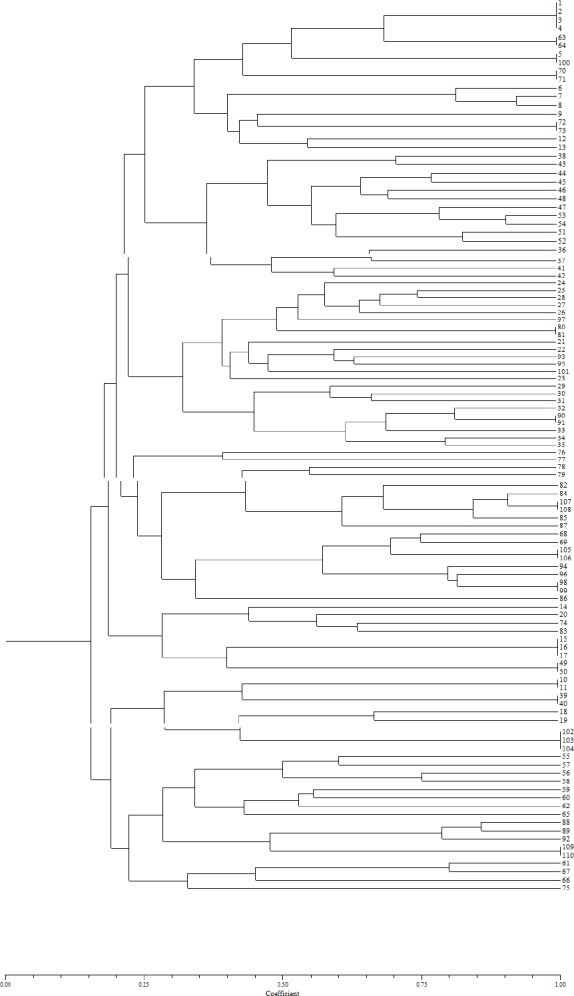
Dendrograms of genomic similarity of 110 CNS strains in Sanandaj hospitals

ERIC Primer, 42-band, with an approximate size between less than 100 bp to bp 1000 reproduce (Figure 3), the clustering results of ERIC-PCR, tested strains were classified in 14 groups. The similarity coefficient between 27 to 50 percent. Maximum strain was in the thirteenth group (Figure 4), a dendrogram that included all profiles was constructed on the basis of the levels of similarity. The thirteenth group with the largest number was formed by 13 CNS strains isolated from Toohid hospital. The eight group with the lowest number contained 2 strains isolated from Toohid hospital. (Table 4)

**Table 4:** Clustering and similarity of CNS isolates and Number of strains in each cluster

Clusters	Dendrogram similarityin clusters	Number of strainsin each cluster		

		Toohid.H	Besat.H	Total
**C1**	42%	7	3	**10**
**C2**	39%	8	0	**8**
**C3**	49%	4	7	11
**C4**	48%	2	2	**4**
**C5**	50%	5	3	**8**
**C6**	38%	3	3	**6**
**C7**	45%	7	2	**9**
**C8**	37%	2	0	**2**
**C9**	44%	6	2	**8**
**C10**	33%	9	0	**9**
**C11**	28%	8	1	**9**
**C12**	29 %	9	0	**9**
**C13**	27%	13	0	**13**
**C14**	34%	4	0	**4**

The agarose gel electrophoresis of the fragments amplified by REP-PCR is shown in figure 3. The profiles generated with primer ERIC contained several bands

**Table 5:** Distribution of genetic and characterization of 14 cluster of 110 CNS clinical isolates

Cluster	number of Profile	percentage of strain	MRCNS	ERCNS	iMLS B phenotypes	Multiplex PCR erm genes	hospital
**C1**	4	9.1%	4.5%	3.6%	2.1%	2.1%	T. H 6.4%
		(10)	5	4	1	1	B. H 2.7%
**C2**	7	7.3%	2.7%	1.8%	0	0	T. H 7.3%
		(8)	3	2			B. H 0.0%
**C3**	11	10%	3.6%	0.0%	0	0	T. H 3.6%
		(11)	4	0			B. H 6.4%
**C4**	4	3.6%	3.6%	0.0%	0	0	T. H 1.8%
		(4)	4	0			B. H 1.8%
**C5**	7	7.3%	5.5%	4.5%	2.1%	2.1%	T. H 4.5%
		(8)	6	5	1	1	B. H 2.7%
**C6**	6	5.5%	4.5%	4.5%	2.1%	2.1%	T. H 2.7%
		(6)	5	5	1	1	B. H 2.7%
**C7**	8	8.2%	6.4%	5.5%	0	0	T. H 6.4%
		(9)	7	6			B. H 1.8 %
**C8**	2	1.7%	1.8%	1.8%	0	0	T. H 1.8 %
		(2)	2	2			B. H 0.0%
**C9**	7	7.3%	3.6%	1.8%	0	0	T. H 5.5%
		(8)	4	2			B. H 1.8%
**C10**	7	8.2%	4.5%	4.5%	0	0	T. H 8.2%
		(9)	5	5			B. H 0.0%
**C11**	6	8.2%	6.4%	5.5%	0	0	T. H 7.3%
		(9)	7	6			B. H 0.9 %
**C12**	5	8.2%	2.7%	4.5%	2.1%	0	T. H 8.2%
		(9)	3	5	1		B. H 0.0 %
**C13**	12	11.8%	5.5%	3.6%	2.1%	2.1%	T. H 11.8%
		(13)	6	4	1	1	B. H 0.0 %
**C14**	4	3.6%	2.7%	1.8%	0	0	T. H 3.6%
		(4)	3	2			B. H 0.0 %
**TOTAL**	90	100%	58.2%	43.6%	10.4%	8.2%	T. H 79.1%
		110	64	48	5	4	B. H 20.9 %

T. H: Toohid hospital; B. H: Beast hospital; CNS: Coagulase negative staphylococci; MRCNS: methicillin -resistant CNS; ERCNS: resistant to erythromycin CNS.

## 4. Discussion

Antibiogram results showed 58.2% strains resistance to Methicillin, 43.6 % resistant to Erythromycin 72.9 % constitutive resistance to Clindamycin and 10.4% inducible resistant to Clindamycin that similar to reported by Jorgensen et al., Ciraj et al., and Goyal et al (Jorgensen, Crawford, McElmeel, & Fiebelkorn, 2004; Ciraj, Vinod, Sreejith, & Rajani, 2009; Goyal, Manchanda, & Mathur, 2004). Many researchers have reported a higher incidence (Rahbar, & Hajia, 2007; Delialioglu, Aslan, Ozturk, Baki, Sen, & Emekdas, 2005; Reinoso, Bettera, Odieno, & Bogni, 2007). While others showed a lower incidence (Ross, Merz, Farkosh, & Carroll, 2005).

27 (24.5%) of the strains Toohid hospital and 4 (3.6%) of the strains Besat hospital were resistance to Methicillin, Erythromycin and cMLS_B_. 5 (4.5%) of 110 sample were iMLS_B_ phenotypes all of them from Toohid hospital that 4 (3.6%) were multiplex PCR positive.

So the different resistance to antimicrobials was found within isolates collected from Besat and Toohid hospitals that these results may indicate differences in Therapeutic measures among these hospitals, particularly, differences in the type drugs and frequency of their use, and so exposure to Patients antimicrobials.

Rep-PCR determination the genetic diversity and also transmission trace of Coagulase negative staphylococci clinical isolates bacterial in the community and hospitals. This is the first study carried out in hospitals of Sanandaj which describes the genetic relationships among CNS isolates hospitalized patients in the intensive care unit and infectious ward of Besat and Toohid hospitals by rep- PCR. As shown in previous reports, rep- PCR was proven to be a highly discriminate and rapid screening method to classify a large number of isolates into clusters (Moosavian, & Darban, 2010; Moosavian, & Wadowsky, 2010; Reinoso, Bettera, Odieno, & Bogni, 2007; Ross, Merz, Farkosh, Carroll, 2005) Most of the strains (11.8 %) were grouped in cluster 13. Genetic heterogeneity among CNS isolates was observed within hospitals. The eighty-seven CNS strains from Toohid hospital were divided into 70 profiles belonged to all clusters and the twenty three isolates from Besat hospital were divided into 20 profiles belonged to all clusters except cluster 2,8,10,12,13,14. Seventy-four (67.3%) of the isolates displayed a single; profile [18(16.4%) Besat, 56(50.9) Toohid] whereas, thirty-six (32.7%) of them showed shared patterns [5 (4.5%) Besat, 31(28.2%) Toohid].

In totally 16 groups obtained with similarity coefficient 100 percent (same profiles within hospitals). Fourteen groups were found in Toohid hospital (strains 63-64; 5,100; 70-71; 72-73; 90-91; 107-108; 105-106; 98-99; 15-17; 49-50; 10-11; 39-40; 102-104; 109-110), two predominant profiles were identified in Besat hospital (strains 1-4; 80-81). Although There wasn’t any same isolates between of two hospitals, isolates obtained from the different wards of hospitals: strains 10, 11 that belonged to intensive care unit and infectious wards hospital Toohid and 1-3, 4 belonged to intensive care unit and infectious wards hospital Besat showed similar genotype which indicate clonal transmission of CNS in wards of hospitals. Among of them only one sample was shown *erm* genes by multiplex PCR that it belonged to infectious ward hospital Toohid it could cause frequent uses of this drugs that it lead to resistance, especially resistance induction, and consequent failure to be treated with clindamycin.

## 5. Conclusion

To conclude, this study showed the genetic relationship in isolates CNS in two hospitals. Most of the isolates showed unique pattern which indicate that the rate of transmission resistant strains are very low in Sanandaj hospitals. The remaining patterns showed similarity in most characteristics which was indicated of similar origin of dissemination.
